# Prevalence of major infections and adverse outcomes among hospitalized. ST-elevation myocardial infarction patients in Florida, 2006

**DOI:** 10.1186/1471-2261-11-69

**Published:** 2011-11-22

**Authors:** Michelle C Nash, Joel A Strom, Elizabeth B Pathak

**Affiliations:** 1 Department of Epidemiology and Biostatistics, College of Public Health, University of South Florida, (13201 Bruce B. Downs Blvd.), Tampa, (33612), USA; 2 Department of Internal Medicine, College of Medicine, University of South Florida, (12901 Bruce B. Downs Blvd.), Tampa, (33612), USA

## Abstract

**Background:**

ST-elevation myocardial infarction (STEMI) patients have risk factors and co-morbidities and require procedures predisposing to healthcare acquired infections (HAIs). As few data exist on the extent and consequences of infections among these patients, the prevalence, predictors, and potential complications of major infections among hospitalized STEMI patients at all Florida acute care hospitals during 2006 were analyzed.

**Methods:**

Sociodemographic characteristics, risk factors, co-morbidities, procedures, complications, and mortality were analyzed from hospital discharge data for 11, 879 STEMI patients age ≥18 years. We used multivariable logistic regression modeling to examine and adjust for multiple potential predictors of any infection, bloodstream infection (BSI), pneumonia, surgical site infection (SSI), and urinary tract infection (UTI).

**Results:**

There were 2, 562 infections among 16.6% of STEMI patients; 6.2% of patients had ≥2 infections. The most prevalent HAIs were UTIs (6.0%), pneumonia (4.6%), SSIs (4.1%), and BSIs (2.6%). Women were at 29% greater risk, Blacks had 23% greater risk, and HAI risk increased 11% with each 5 year increase in age. PCI was the only protective major procedure (OR 0.81, 95% CI, 0.69-0.95, p < .05). HAI lengthened hospital stays. STEMI patients with a BSI were almost 5 times more likely (31.3% vs. 6.5%, p < .0001), and those with pneumonia were 3 times more likely (19.6% vs. 6.5%, p < .0001) to die before discharge.

**Conclusions:**

The protective effect of PCI on risk of infection is likely mediated by its many benefits, including reduced length of hospitalizations.

## Background

Approximately two million health care acquired infections (HAIs) occur annually (affecting 5-10% of all inpatients and 25% of intensive care unit (ICU) patients), contributing to an estimated 90, 000 deaths and $4.5 to $5.7 billion dollars in excess healthcare expenditures [1,2]. HAI patients average five times the length of stay (LOS) and treatment cost and six times the in-hospital mortality versus those without HAIs [3]. Over 80% of HAIs belong to four categories: urinary tract infection (UTI) (35% of all infections), surgical site infection (SSI) (20%), bloodstream infection (BSI) (15%), and pneumonia (15%) [2]. BSIs and pneumonia most significantly increase mortality [2]. UTIs, despite their prevalence, result in the lowest additional treatment costs and excess mortality. Risks of hospital acquired UTI include: female sex, non-white ethnicity, co-morbidities, unit of admission, indwelling catheters, invasive procedures, and mechanical ventilation [4]. Mechanical ventilator use is also strongly associated with development of pneumonia [2], while indwelling intravascular catheters, implanted medical devices, LOS in ICU or surgical ward, and invasive procedures are associated with bacteremias [5].

Few studies have examined predictors of HAIs among acute myocardial infarction (AMI) patients, especially ST-elevation myocardial infarction (STEMI) [6]. Among cardiac care unit (CCU) occupants, pneumonia, BSIs, and UTIs were strongly associated with invasive device implantation (i.e. mechanical ventilator, catheter). However, overall incidence of CCU-acquired HAIs was less than in other ICUs, attributable to less frequent use of invasive devices [7]. Reperfusion therapy may cause HAI, by frequent use of invasive devices and procedures and procedure-related complications, e.g. contrast-induced nephropathy. While infrequently utilized for STEMI patients, surgical revascularization increases the risk for surgical site infections, pneumonia, and UTIs [8,9].

To define the prevalence and clinical impact of HAI in STEMI patients, we examined the prevalence, predictors, and potential complications of major infections among hospitalized STEMI patients at all acute care hospitals in Florida during 2006 (before the October 2007 Medicare mandate to report "present on admission" data for infections), thereby providing baseline population-based and unselected prevalence data on infections in a high-risk patient population.

## Methods

### Study Population and Data Sources

All adults age ≥18 years with a primary diagnosis of STEMI admitted to non-Veterans Administration acute care hospitals in 2006 were included. Surveillance data was obtained from the Florida Agency for Health Care Administration Hospital Discharge Database, which captures 100% of hospital discharges. Patient variables analyzed: age, race/ethnicity, gender, insurance type, type and source of admission, admitting diagnosis, principal diagnosis, secondary diagnoses (up to 30 per patient), procedures (up to 30 per patient), length of stay, and vital status at discharge. Diagnoses and procedures were coded using the International Classification of Diseases (9^th ^Revision), Clinical Modification (ICD-9-CM). The University of South Florida IRB granted the study exempt status.

A total of 14, 148 patient admissions carried a principal diagnosis of STEMI in 2006, identified by the principal diagnosis ICD-9-CM codes 410.0 to 410.6 and 410.8, with a fifth digit of 0 or 1. After excluding transfers (n = 2, 269), because we could not link separate hospital records, 11, 879 STEMI patients remained.

### Variable Definitions

Infections were identified by secondary diagnosis ICD-9-CM codes representing conditions listed in *CDC/NHSN surveillance definition of health care-associated infection and criteria for specific types of infections in the acute care setting *(2008) [10]. We added to a list of HAI ICD-9-CM codes compiled by Sherman et al. (2006) [11] by completing our own review of ICD-9-CM infection codes. Patient risk factors, co-morbidities, and procedures were identified from the thirty ICD-9-CM procedure and secondary diagnosis codes.

Based on CDC surveillance guidelines, we grouped infection codes into the following categories: (1) BSI, (2) pneumonia, (3) SSI, (4) UTI, (5) central nervous system illness, (6) gastrointestinal illness, (7) lower respiratory tract infection, (8) skin and soft tissue infection, (9) bone and joint infection, (10) cardiovascular infection, and (11) ear, eye, nose, and throat infection. Only categories 1-4 included enough patients to warrant separate modeling of predictors. Categories 6-11 were collapsed into an "other infection" group (there were no cases in category 5), and included patients with secondary diagnosis ICD-9-CM codes for site unspecified infections. Patients with ≥2 infections could be included in more than one group.

Patient characteristics analyzed were age, sex, race/ethnicity, and socioeconomic status. Asians/Pacific Islanders, American Indians/Alaskan Natives were grouped with whites due to their very small numbers. Insurance type (Medicaid, other state/government, self pay, underinsured, and charity) was a proxy for low income. Risk factors analyzed were cigarette smoking, alcohol, and drug abuse. Our analysis included six serious co-morbidities (chronic bronchitis, COPD, diabetes mellitus, chronic kidney diseases, cardiomyopathy, and valve disorders), and the following procedures: cardiac catheterization, percutaneous coronary intervention (PCI), cardiac surgical procedures, indwelling arterial or venous catheter, pacemakers and/or implantable defibrillators, dialysis, and blood transfusion. Hospital LOS was the total number of days between admission and discharge. ICU or CCU stay was a dichotomous variable (yes/no) based on the presence or absence of ICU/CCU financial charges in the discharge record.

### Statistical Analyses

We evaluated the proportion of STEMI patients with any infection and for each category, along with the total number of infections present and the prevalence of multiple infections. We used multivariable logistic regression modeling to examine and adjust for multiple potential predictors of any infection, BSI, pneumonia, SSI, and UTI. The 9, 910 patients with no diagnosed infection comprised the comparison group for all models.

Several important co-morbidities (acute renal failure, heart failure), procedures (mechanical ventilation, Swan-Ganz catheter), and outcomes (admission to the ICU/CCU, hospital LOS), important potential complications of infection, were not included in the predictive models because the temporal relationship to the onset of infection could not be determined, e.g. whether ICU stay resulted in or was a consequence of an infection. The prevalence of each potential infectious complication was compared to the prevalence among non-infected patients using a chi-square test of homogeneity. The in-hospital mortality for each infection group was compared to non-infected STEMI patients. We used SAS version 9.1.3 (SAS Institute, Cary, North Carolina) for all analyses.

## Results

There were 2, 562 infections among 1, 969 (16.6%) STEMI patients; 739 (6.2%) patients had ≥2 infections. UTIs (6.0%) were most prevalent, followed by pneumonia (4.6%), SSIs (4.1%), and BSIs (2.6%). Their relative frequencies were 36.1%, 27.7%, 24.7%, and 15.7%, respectively. Lower respiratory tract, cardiovascular, bone and joint, eye, ear, nose, and throat, gastrointestinal, skin and soft tissue, and non-specific bacterial infections (other infection) comprised 4.2% of patients (Table 1). Thirty-seven (0.3%) patients had an intra-cardiac or pericardial infection. Overall, STEMI patients were older, more likely to be white and male, and a nearly one-third smoked (28.9%) and had diabetes (27.1%). The most common procedure was cardiac catheterization (66.9%), followed by PCI (59.0%). Infected patients were older, less likely to be smokers and receive PCI, and had more co-morbidities than patients with no infection (Table 2). The majority of infected STEMI patients underwent at least one minor or major procedure.

**Table 1:** Infections among hospitalized STEMI patients (n = 11, 879)* in Florida, 2006.

Infection Categories^†^	Frequency of Infection^‡ ^% (n)
Infected STEMI Patients	16.6 (1, 969)

Blood Stream Infection (BSI)	2.6 (310)

Any additional infection	75.0 (233)

No additional infection	25.0 (77)

Pneumonia	4.6 (550)

Any additional infection	41.0 (224)

No additional infection	59.0 (326)

Surgical Site Infection (SSI)	4.1 (491)

Any additional infection	30.0 (145)

No additional infection	70.0 (346)

Urinary Tract Infection (UTI)	6.0 (708)

Any additional infection	35.0 (248)

No additional infection	65.0 (460)

All Other Infections^§^	4.2 (503)

*Excludes patients who were transferred to another hospital.

†Patients can appear in more than one infection category.

‡There were a total of 2, 562 infections.

§Includes lower respiratory, gastrointestinal, cardiovascular, bone and joint, and ear, eye, nose & throat, skin and soft tissue, and site unspecified bacterial infections.

**Table 2:** Characteristics of hospitalized STEMI patients (n = 11, 879)* by infection status, Florida 2006.

	All STEMI Patients n = 11, 879 *% (n)*	Non-Infected STEMI Patients n = 9, 910 *% (n)*	Blood Stream Infection^†^ n = 310 *% (n)*	Pneumonia n = 550 *% (n)*	Surgical Site Infection n = 491 *% (n)*	Urinary Tract Infection n = 708 *% (n)*	All Other Infections^‡^ n = 503 *% (n)*
**Patient Sociodemographic** **Characteristics^**							

**Age**							

Age 18-34 Years	0.9 (107)	1.0 (100)	No cases**	No cases**	0.6 (3) ‡‡	0.3 (2)**	0.8 (4)**
Age 35-44 Years	6.2 (735)	6.6 (658)	4.9 (15)	2.4 (13)	5.9 (29)	1.4 (10)	6.4 (32)
Age 45-54 Years	18.7 (3057)	20.0 (1984)	10.1 (31)	3.6 (47)	20.6 (101)	5.4 (38)	14.5 (73)
Age 55-64 Years	23.9 (2837)	24.9 (2471)	19.5 (60)	19.6 (108)	22.8 (112)	14.0 (99)	19.7 (99)
Age 65-74 Years	21.7 (2573)	21.4 (2116)	24.7 (76)	24.9 (137)	25.3 (124)	21.2 (150)	22.3 (112)
Age 75-84 Years	18.5 (2196)	17.0 (1680)	26.3 (81)	28.0 (154)	19.1 (94)	34.0 (241)	23.7 (119)
Age 85+ Years	10.2 (1216)	9.1 (901)	14.6 (45)	16.6 (91)	5.7 (28)	23.7 (168)	12.7 (64)

**Sex**							

Female	33.7 (4001)	31.8 (3149)	38.6 (119) ††	38.6 (212) ††	30.4 (149)	59.5 (421)**	42.9 (216)**
Male	66.3 (7878)	68.2 (6761)	61.4 (189)	61.5 (338)	69.7 (342)	40.5 (287)	57.1 (287)

**Race/Ethnicity**							

White	83.1 (9868)	83.3 (8256)	78.3 (241)	81.8 (450)	81.5 (400)	82.2 (582)	82.3 (414)
Black	6.7 (798)	6.5 (640)	9.1 (28)	7.6 (52)	9.2 (45)	8.2 (58)	7.4 (37)
Hispanic	10.2 (1213)	10.2 (1014)	12.7 (39)	10.6 (58)	9.4 (46)	9.6 (68)	10.3 (52)

**Income by Proxy**							

Low income (Medicaid, self-pay, uninsured)	16.7 (1979)	17.3 (1711)	13.0 (40) ‡‡	10.6 (58)**	19.6 (96)	9.5 (67)**	15.9 (80)
All other insurance^§^	83.3 (9900)	82.7 (8199)	87.0 (268)	89.5 (492)	80.5 (395)	90.5 (641)	84.1 (423)

**Patient Risk Factors**							

Cigarette smoking	28.9 (3430)	30.6 (3030)	14.9 (46)**	22.4 (123)**	24.4 (120) ††	12.0 (85)**	23.7 (119) ††
Alcohol abuse	3.0 (352)	2.8 (273)	4.6 (14)	5.3 (29) ††	4.9 (24) ††	2.0 (14)	5.8 (29)**
Drug abuse	2.4 (287)	2.4 (237)	2.3 (7)	2.2 (12)	3.3 (16)	1.6 (11)	4.2 (21) ‡‡

**Patient Co-Morbidities**							

Chronic bronchitis	2.7 (317)	2.2 (213)	6.8 (21)**	10.0 (55)**	4.3 (21) ††	5.1 (36)**	4.0 (20) ††
Chronic obstructive pulmonary disease (COPD)	16.2 (1921)	14.0 (1382)	28.3 (87)**	30.7 (169)**	20.8 (102)**	24.0 (170)**	37.0 (186)**
Diabetes	27.1 (3222)	25.5 (2527)	35.1 (108) §§	34.2 (188)**	32.4 (159) ††	38.6 (273)**	37.2 (187)**
Chronic kidney diseases^| |^	9.6 (1145)	7.7 (764)	29.9 (92)**	22.7 (125)**	13.4 (66)**	23.2 (164)**	17.1 (86)**
Cardiomyopathy	6.3 (755)	5.5 (546)	16.9 (52)**	12.7 (70)**	9.8 (48)**	11.0 (78)**	11.9 (60)**
Valve disorders	10.1 (1203)	9.1 (902)	21.4 (66)**	16.0 (88)**	11.8 (58) ‡‡	16.2 (115)**	17.9 (90)**

**Patient Procedures**							

Cardiac catheterization	66.9 (7941)	67.7 (6710)	59.4 (184) ††	57.1 (314)**	80.7 (396)**	54.2 (384)**	65.4 (329)
Percutaneous coronary intervention (PCI)	59.0 (7009)	61.2 (6065)	41.6 (128)**	44.0 (242)**	62.1 (305)	41.0 (290)**	47.9 (241)**
Cardiac surgical procedures	9.8 (1168)	8.4 (830)	24.4 (75)**	18.6 (102)**	19.4 (95)**	16.4 (116)**	24.1 (121)**
Indwelling arterial or venous catheter	6.0 (707)	3.6 (360)	45.2 (140)**	26.6 (146)**	18.1 (89)**	18.9 (134)**	20.9 (105)**
Pacemaker^#^	2.3 (275)	2.1 (210)	3.6 (11)	4.2 (23) ††	4.9 (24)**	2.3 (16)	3.6 (18) ‡‡
Dialysis	1.5 (172)	0.7 (68)	16.5 (51)**	6.9 (38)**	6.5 (32)**	5.1 (36)**	6.6 (33)**
Blood transfusion	6.7 (800)	5.1 (507)	26.6 (82)**	15.3 (84)**	15.3 (75)**	17.5 (124)**	15.5 (78)**
Patients with none of the above procedures	26.7 (3169)	26.5 (2630)	21.0 (65) ‡‡	30.4 (167) ‡‡	11.0 (54)**	34.9 (247)**	23.9 (120)

*Excludes patients who were transferred to another hospital.

** *p *< .0001; †† *p *< .01; ‡‡ *p *< .05; §§ *p *= .0001

†Patients can appear in more than one infection category.

‡Includes lower respiratory, gastrointestinal, cardiovascular, bone and joint, ear, eye, nose & throat, skin and soft tissue, and site unspecified bacterial infections.

§Includes Medicare, commercial insurance, worker's compensation, CHAMPUS, VA, and unspecified.

| |Includes hypertensive chronic kidney disease, end-stage renal disease (ESRD), unspecified chronic kidney disease and unspecified chronic renal failure.

#Includes temporary/permanent pacemakers and implantable defibrillators.

^Chi-square tests of homogeneity were conducted to compare patient characteristics for each infection to non-infected STEMI patients (excluding all patients).

After multivariable adjustment, all other risk factors and co-morbidities examined were associated with increased risk of infection, except for cigarette smoking (OR 0.76, 95% CI 0.66-0.87, p < .01) (Table 3). Women and Blacks had a 29% and 23% greater risk, respectively, while the risk of infection increased 11% with each 5 year increase in age. Most procedures conveyed an increased risk - the notable exception was the lower risk associated with PCI (OR 0.81, 95% CI, 0.69-0.95, p < .01).

**Table 3:** Multivariable adjusted odds ratios for predictors of infection among STEMI Patients (n = 11, 879)*, ^† ^at acute care hospitals in Florida, 2006.

	All Infections n = 1, 969 Odds-Ratio (95% CI)	Blood Stream Infection^3^ n = 310 Odds Ratio (95% CI)	Pneumonia^‡^ n = 550 Odds Ratio (95% CI)	Surgical Site Infection^‡^ n = 491 Odds Ratio (95% CI)	Urinary Tract Infection^‡^ n = 708 Odds Ratio (95% CI)
**Patient Characteristics**					

**Age**					

Each 5 year increase	1.11 (1.00 - 1.14) ‡‡	1.06 (0.95 - 1.17)	1.21 (0.14 - 1.28)	0.99 (0.94 - 1.04)	1.27 (1.21 - 1.33)**

**Sex**					

Female	1.29 (1.15 - 1.44)**	0.71 (0.43 - 1.21)	0.83 (0.64 - 1.06)	0.80 (0.62 - 1.04)	2.46 (1.99 - 3.03)**

**Race/Ethnicity**					

Black race	1.23 (1.00 - 1.51) ‡‡	1.27 (0.54 - 2.97)	1.19 (0.75 - 1.90)	1.44 (0.98 - 2.11)	1.01 (0.67 - 1.52)
Hispanic ethnicity	1.05 (0.88 - 1.25)	1.92 (0.99 - 3.73)	0.98 (0.65 - 1.47)	0.84 (0.57 - 1.22)	1.02 (0.70 - 1.42)

**Income by Proxy**					

Low income (Medicaid, self-pay, uninsured)	1.14 (0.97 - 1.34)	1.36 (0.69 - 2.71)	1.07 (0.73 - 1.58)	1.30 (0.97 - 1.73)	1.13 (0.78 - 1.64)

**Patient Risk Factors**					

Cigarette smoking	0.76 (0.66 - 0.87) ††	0.62 (0.32 - 1.22)	1.16 (0.86 - 1.56)	0.70 (0.53 - 0.91) ††	0.66 (0.48 - 0.82) ‡‡
Alcohol abuse	1.73 (1.30 - 2.31) ††	0.94 (0.20 - 4.44)	2.31 (1.33 - .01) ††	1.67 (0.96 - 2.90)	0.93 (0.37 - 2.36)
Drug abuse	1.55 (1.09 - 2.20) ‡‡	1.80 (0.37 - 8.80)	0.64 (0.22 - 1.84)	1.02 (0.52 - 2.00)	1.38 (0.48 - 3.84)

**Patient Co-Morbidities**					

Chronic bronchitis	2.39 (1.84 - 3.09)**	2.56 (0.87 - 7.52)	4.30 (2.82 - .56)**	1.17 (0.54 - 2.53)	1.47 (0.85 - 2.53)
Chronic obstructive pulmonary disease (COPD)	2.03 (1.79 - 2.30)**	1.80 (1.03 - 3.14) ‡‡	2.28 (1.75 - .96)**	1.22 (0.90 - 1.65)	1.57 (1.24 - 2.00) ††
Diabetes	1.33 (1.19 - 1.49)**	0.94 (0.56 - 1.58)	1.10 (0.85 - 1.42)	1.24 (0.98 - 1.58)	1.59 (1.30 - 1.96)**
Chronic Kidney Diseases^§^	1.47 (1.26 - 1.72)**	1.72 (0.92 - 3.23)	1.43 (1.04 - .98) ‡‡	0.85 (0.54 - 1.32)	1.64 (1.26 - 2.14) ††
Cardiomyopathy	1.50 (1.25 - 1.80)**	1.55 (0.75 - 3.22)	1.20 (0.80 - 1.81)	1.24 (0.81 - 1.91)	1.26 (0.89 - 1.71)
Valve disorders	1.17 (1.01 - 1.37) ‡‡	2.22 (1.26 - 3.92) ††	1.22 (0.88 - 1.68)	0.94 (0.64 - 1.39)	0.91 (0.69 - 1.22)

**Patient Procedures**					

Cardiac catheterization	1.10 (0.93 - 1.30)	0.51 (0.25 - 1.04)	0.79 (0.55 - 1.15)	2.89 (1.98 - 4.22)**	1.07 (0.77 - 1.47)
Percutaneous coronary intervention (PCI)	0.81 (0.69 - 0.95) ††	1.05 (0.53 - 2.08)	0.85 (0.59 - 1.21)	0.63 (0.46 - 0.88) ††	0.85 (0.63 - 1.16)
Cardiac surgical procedures	1.53 (1.28 - 1.84)**	1.42 (0.65 - 3.14)	1.54 (1.02 - .33)#	0.66 (0.43 - 1.01)	1.83 (1.28 - 2.62) ††
Indwelling arterial or venous catheter	3.90 (3.29 - 4.63)**	6.99 (3.93 - 12.41)**	3.93 (2.79 - .55)**	1.98 (1.30 - 3.03) ††	1.69 (1.17 - 2.43) ††
Pacemaker^| |^	1.02 (0.74 - 1.40)	0.63 (0.14 - 2.80)	1.41 (0.76 - 2.58)	1.43 (0.79 - 2.57)	0.61 (0.30 - 1.23)
Dialysis	3.40 (2.40 - 4.82)**	6.97 (2.81 - 17.26)**	3.55 (1.76 - .09) ††	2.61 (1.10 - .17) ‡‡	2.24 (1.19 - 4.19) ‡‡
Blood Transfusion	1.64 (1.37 - 1.96)**	2.10 (1.07 - 4.11) ‡‡	0.90 (0.58 - 1.40)	1.62 (1.08 - .45) ‡‡	1.94 (1.43 - 2.64)**

*Excludes patients who were transferred to another hospital.

** *p *< .0001; †† *p *< .01; ‡‡ *p *< .05; §§ *p *= .0001

†All models compared to non-infected STEMI patients and are adjusted for all other variables in the table.

‡Patients can appear in more than one infection category.

§Includes hypertensive chronic kidney disease, end-stage renal disease (ESRD), unspecified chronic kidney disease and unspecified chronic renal failure.

| |Includes temporary/permanent pacemakers and implantable defibrillators.

### Bloodstream Infections

BSIs were the most serious but least common type of HAI (Table 3). Valve disease (OR 2.22, 95% CI 1.26-3.92, p < .01) and COPD (OR 1.80, 95% CI 1.03-3.14, p < .05) conveyed particular risk. A trend toward elevated prevalence was observed among Hispanics, older patients, and those with chronic kidney diseases (including ESRD) (p = n.s.). Procedures with the strongest association were an indwelling arterial or venous catheter (OR 6.99, 95% CI 3.93-12.41, p < .0001), dialysis (OR 6.97, 95% CI 2.81-17.26, p < .0001), and blood transfusion (OR 2.10, 95% 1.07-4.11, p < .05). BSI sufferers were more likely than non-infected patients to develop acute renal failure (51.3% vs. 5.3%, p < .0001) or heart failure (58.8% vs. 18.0%, p < .0001), and were almost 5 times as likely to die before discharge (31.3% vs. 6.5%, p < .0001) (Table 4).

**Table 4:** Potential complications of infection among hospitalized STEMI patients (n = 11, 879)* in Florida, 2006.

	Non-Infected STEMI Patients n = 9, 910 *% (n)*	Blood Stream Infection n = 310^†^ *% (n)*	Pneumonia n = 550 *% (n)*	Surgical Site Infection n = 491 *% (n)*	Urinary Tract Infection n = 708 *% (n)*	All Other Infections n = 503^‡^ *% (n)*
**Potential Complications of Infection^§^**						

Acute renal failure	5.3 (526)	51.3 (158)**	30.6 (168)**	15.5 (76)**	28.8 (204)**	23.1 (116)**
Heart failure	18.0 (1776)	58.8 (181)**	63.5 (349)**	26.9 (132)**	50.6 (358)**	46.9 (236)**
Swan-Ganz catheter	1.3 (129)	8.8 (27)**	5.1 (28)**	3.9 (19)**	4.0 (28)**	5.0 (25)**
Mechanical ventilation	5.4 (537)	14.9 (46)**	13.1 (72)**	21.2 (104)**	8.5 (60)**	25.1 (126)**
Intensive care unit stay	64.0 (6339)	69.8 (215) ‡‡	69.3 (381) ‡‡	68.2 (335) ‡‡	70.6 (500)^	67.8 (341)
Length of stay 7+ days	15.5 (1535)	75.7 (233)**	65.5 (360)**	44.8 (220)**	57.6 (408)**	61.6 (310)**
In-hospital mortality	6.5 (640)	31.3 (97)**	19.6 (108)**	7.9 (39)	11.0 (78)**	7.4 (37)

* Excludes patients who were transferred to another hospital.

** *p *< .0001; †† *p *< .01; ‡‡ *p *< .05; §§ *p *= .0001

† Patients can appear in more than one infection category.

‡ Includes lower respiratory, gastrointestinal, cardiovascular, bone and joint, ear, eye, nose & throat, skin and soft tissue, and site unspecified bacterial infections.

§ Chi-square tests of homogeneity were conducted to compare potential complicating factors of each infection to non-infected STEMI patients.

### Pneumonia

Pneumonia risk increased with alcohol abuse (OR 2.31, 95% CI 1.33-4.01, p < .01), chronic bronchitis (OR 4.30, 95% CI 2.82-6.56, p < .0001), and COPD (OR 2.28, 95% CI 1.75-2.96, p < .0001), but not with sociodemographic characteristics (Table 3). Indwelling intravascular catheters (OR 3.93, 95% CI 2.79-5.55, p < .0001), dialysis (OR 3.55, 95% CI 1.76-7.09, p < .01), and cardiac surgical procedures (OR 1.54, 95% CI 1.02-2.33, p < .05) were associated with an increased risk. Although mechanical ventilation has been reported to convey a strong risk for development of pneumonia, [12] only 13.1% of the STEMI patients with pneumonia were ventilated during their hospital stay (Table 4). Pneumonia was complicated more frequently by acute renal failure (30.6% vs. 5.3%, p < .0001), heart failure (63.5% vs. 18.0%, p < .0001), and patients were 3 times as likely to in-hospital (19.6% vs. 6.5%, p < .0001).

### Surgical Site Infections

Cardiac catheterization (OR 2.89, 95% CI 1.98-4.22, p < .0001) was the strongest predictor of SSI, followed by dialysis (OR 2.61, 95% CI 1.10-6.17, p < .05), indwelling intravascular catheters (OR 1.98, 95% CI 1.30-3.03, p < .05), and blood transfusion (OR 1.62, 95% CI 1.08-2.45, p < .05). However, PCI was associated with a lower risk of SSI (OR 0.63, 95% CI 0.46-0.88, p < .01). Except for the lower risk of infection associated with cigarette smoking (OR 0.70, 95% CI 0.53-0.91, p < .01), co-morbidities and risk factors did not impact risk of SSI. We observed a moderate trend (p = n.s.) toward increased risk of SSI among Black and low income patients. Overall, the prevalence of potential complications of SSIs was lower than for BSIs and pneumonia (Table 4), although these patients were almost 4 times more likely to be mechanically ventilated than non-infected patients (21.2% vs. 5.4%, p < .0001). In-hospital mortality was 21% more likely among these patients (7.9% vs. 6.5%, p = n.s.).

### Urinary Tract Infections

UTI, the most common infection complicating STEMI, occurred in women more frequently than men (OR 2.46, 95% CI 1.99-3.03, p < .0001) (Table 3). Each 5-year increase in patient age increased its risk by 27% (OR 1.27, 95% CI 1.21-1.33, p < .0001). Co-morbidities and procedures associated with a higher prevalence of UTIs were: chronic kidney disease (OR 1.64, 95% CI 1.26-2.14, p < .01), diabetes (OR 1.59, 95% CI 1.30-1.96, p < .0001), COPD (OR 1.57, 95% CI 1.24-2.00, p < .01), dialysis (OR 2.24, 95% CI 1.19-4.19, p < .05), blood transfusion (OR 1.94, 95% CI 1.43-2.64, p < .0001), cardiac surgical procedures (OR 1.83, 95% CI 1.28-2.62, p < .01), and an indwelling intravascular catheter (OR 1.69, 95% CI 1.17-2.43, p < .01). UTI patients had a much higher prevalence of acute renal failure (28.8% vs. 5.3%, p < .0001) and heart failure (50.6% vs. 18.0%, p < .0001), and had a 69% higher risk of in-hospital death (11.0% vs. 6.5%, p < .0001) (Table 4).

## Discussion

Our study revealed that 16.6% of STEMI patients hospitalized in Florida experienced HAI, approaching the 20.6% prevalence for ICU-acquired infections reported by Vincent et al. [13] and over three times the 5% rate reported in studies of AMI [14] and CCU patients [7,15]. Consistent with ICU patients with nosocomial infections [13,16], infected STEMI patients experienced prolonged hospital stays and were more likely to die prior to discharge. After multivariable adjustment, important factors associated with infection included older age, female gender, Black race, alcohol or drug use, and several serious co-morbidities. Older age, female gender, diabetes, and chronic kidney diseases were all strongly associated with the risk of UTI, consistent with an increased prevalence of asymptomatic bacteriuria and UTIs reported in elderly women with those comorbidities [17]. Urinary tract catheterization can potentiate the risk for serious and potentially life threatening UTIs in these patients.

Cigarette smoking was associated with a lower risk of HAI, despite well-known deleterious actions of cigarette smoke [18]. A possible explanation is that AMI patients who smoke tend to be younger than non-smokers and may possess fewer comorbidities, reducing their likelihood of infection [19]. This observed lower risk of infection in smokers may contribute to the reduced mortality rates reported by us [20] and others [21], especially since the majority of STEMI patients who smoked were younger than those who did not smoke.

Major procedures, especially dialysis, cardiac surgical procedures, blood transfusions, and indwelling arterial or venous catheters were associated with a higher prevalence of infection. While insertion, catheter number, and implantation duration of indwelling catheters increase the risk of both insertion site and systemic infections, catheter use is also a marker of the severity of the patient's underlying condition.

Significantly, PCI was found to be associated with a much lower risk of HAI, particularly SSI. In contrast, cardiac catheterization was associated with an increased risk of SSI. While the risk of infection due to both cardiac catheterization and PCI is low (< 1%), a greater risk occurs with PCI, especially when complicated [22,23]. This paradoxical effect may be explained by cardiac catheterization being a surrogate marker for other HAI-increasing procedures, e.g. cardiac surgical procedures, or the complications related to non-reperfusion therapy. Only 4.2% of the total cohort received thrombolysis. Demographic and comorbid factors could confound the association of catheterization with HAI, e.g. approximately 64% of those not receiving catheterization were ≥65 years old compared to 42% of those who did, and all analyzed co-morbidities occurred slightly more frequently (60.2% vs. 39.8%) in non-catheterized patients. These differences were addressed in the regression analysis. Thus, the lower risk associated with PCI could reflect the over-riding benefit of this STEMI treatment. PCI patients suffer less myocardial damage, resulting in improved hemodynamic and clinical states, leading to shorter ICU/CCU stays and earlier hospital discharge, contributing to reduced HAI exposure.

### Study Limitations

Limitations of our study include the potential for misclassification of both secondary diagnosis and procedures codes. Sherman et al. reported that administrative data reviews were not as accurate as targeted active surveillance at identifying HAIs [11]. Major procedures performed in surgical wards are likely to be correctly ICD-9-CM coded; however, minor procedures performed in patients' rooms or in radiology departments are often inaccurately coded or missing [24]. Consequently, we could not evaluate the prevalence of indwelling urinary catheters, a minor procedure rarely reported. In our 2006 dataset, "present on admission" indicator variables were incomplete and could not be analyzed. These coding inaccuracies may persist, because as of October 2007 Medicare no longer reimburses hospitals for treatment costs incurred by HAIs [25]. Thus, the prevalence of HAIs is likely underestimated.

Our study could not definitively establish a temporal association among co-morbidities, procedures, and development of infections. This limitation should be addressed in future studies with more detailed clinical data, preferably by medical chart review. However, even with chart review, temporality may often be difficult to establish, because the detailed physical exams and multiple tissue cultures necessary to establish "present on admission" infections may not routinely be completed for STEMI patients in many hospitals. This limitation particularly applies to UTIs, which are not rare in community-dwelling populations and are often asymptomatic in elderly women [17], but is less problematic than for other types of infections. Finally, any selection biases of patients for PCI and the relationship of the timing of PCI to admission could not be determined.

## Conclusions

We conclude that: 1) Approximately one sixth of STEMI patients hospitalized in Florida develop an HAI, a prevalence rate approaching that for ICU patients, often resulting in prolonged hospital stays and adverse clinical outcomes including in-hospital mortality; 2) BSIs and pneumonia were associated with the greatest likelihood of prolongation of hospital stay and in-hospital mortality; and 3) PCI was associated with reduced risk of overall infection, especially SSI. While current STEMI treatment guidelines do not emphasize risk of HAI [26,27], improved surveillance, prevention, and treatment for high risk patients may reduce the risk. Despite limitations inherent in this type of study, the lower risk of infection associated with PCI provides additional support for the use of primary PCI for STEMI therapy.

## Competing interests

The authors declare that they have no competing interests.

## Authors' contributions

EB and MN completed the data analysis, but each author contributed equally to the research design and implementation. All authors have read and approved the final manuscript.

## Pre-publication history

The pre-publication history for this paper can be accessed here:

http://www.biomedcentral.com/1471-2261/11/69/prepub
